# *tert*-Butyl 4-formyl-1*H*-imidazole-1-carboxyl­ate

**DOI:** 10.1107/S1600536810029247

**Published:** 2010-07-31

**Authors:** Jun-Tao Kang, Zhi-Gang Li, Jing-Wei Xu, Yang Wei

**Affiliations:** a The State Key Laboratory of Electroanalytical Chemistry, Changchun Institute of Applied Chemistry, Chinese Academy of Sciences, Changchun 130022, People’s Republic of China

## Abstract

In the crystal structure of the title compound, C_9_H_12_N_2_O_3_, weak inter­molecular C—H⋯O hydrogen bonds link the mol­ecules into chains. Further weak C—H⋯O hydrogen bonds together with π–π inter­actions [centroid–centroid distance = 3.672 (4) Å] between neighbouring chains lead to a double-chain structure propagating in [100].

## Related literature

For uses of imidazole direvatives, see: Matuszak *et al.* (1976 ▶), Verras *et al.* (2004 ▶). For the synthesis of the title compound, see: Metobo *et al.* (2006 ▶).
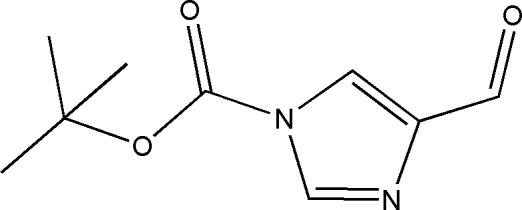


## Experimental

### 

#### Crystal data

C_9_H_12_N_2_O_3_
*M* *_r_* = 196.21
Triclinic, 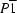

*a* = 5.972 (3) Å
*b* = 7.173 (7) Å
*c* = 12.164 (11) Å
α = 79.630 (16)°
β = 86.620 (15)°
γ = 89.326 (15)°
*V* = 511.7 (8) Å^3^
*Z* = 2
Mo *K*α radiation
μ = 0.10 mm^−1^
*T* = 293 K
0.14 × 0.11 × 0.03 mm

#### Data collection

Bruker APEX CCD area detector diffractometer
Absorption correction: multi-scan (*SADABS*; Bruker, 2001 ▶) *T*_min_ = 0.987, *T*_max_ = 0.997
2633 measured reflections
1769 independent reflections
915 reflections with *I* > 2σ(*I*)
*R*_int_ = 0.022

#### Refinement

*R*[*F* ^2^ > 2σ(*F* ^2^)] = 0.062
*wR*(*F* ^2^) = 0.149
*S* = 0.99
1769 reflections
130 parameters
H-atom parameters constrained
Δρ_max_ = 0.17 e Å^−3^
Δρ_min_ = −0.17 e Å^−3^

### 

Data collection: *SMART* (Bruker, 2007 ▶); cell refinement: *SAINT-Plus* (Bruker, 2007 ▶); data reduction: *SAINT-Plus*; program(s) used to solve structure: *SHELXS97* (Sheldrick, 2008 ▶); program(s) used to refine structure: *SHELXL97* (Sheldrick, 2008 ▶); molecular graphics: *SHELXTL* (Sheldrick, 2008 ▶); software used to prepare material for publication: *SHELXTL*.

## Supplementary Material

Crystal structure: contains datablocks global, I. DOI: 10.1107/S1600536810029247/su2164sup1.cif

Structure factors: contains datablocks I. DOI: 10.1107/S1600536810029247/su2164Isup2.hkl

Additional supplementary materials:  crystallographic information; 3D view; checkCIF report

## Figures and Tables

**Table 1:** Hydrogen-bond geometry (Å, °)

*D*—H⋯*A*	*D*—H	H⋯*A*	*D*⋯*A*	*D*—H⋯*A*
C2—H2⋯O3^i^	0.93	2.36	3.251 (5)	160
C9—H9*C*⋯O1^ii^	0.96	2.65	3.531 (5)	153

Symmetry codes: (i) 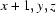; (ii) 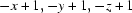.

**Fig. 1.**
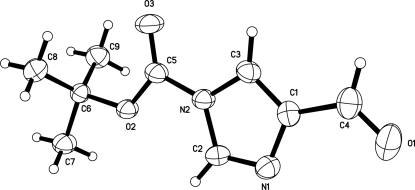
A view of the molecular structure of the title compound, with the atom-labeling scheme and 30% probability displacement ellipsoids.

**Fig. 2.**
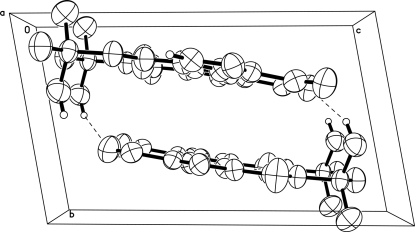
Perspective view along the a-axis of the crystal packing of the title compound, showing the C-H···O interactions as dashed lines [H-atoms not involved in the motif shown have been omitted for clarity].

## supplementary crystallographic information

### Comment

Imidazole derivatives are very useful compounds (Matuszak *et al.*, 1976; Verras *et al.*, 2004), and herein we report on the molecular and crystal structure of the title compound, illustrated in Fig. 1.

In the crystal packing of the title compound (Fig. 2), C2—H2···O3 hydrogen bonds involving adjacent molecules lead to the formation of a one-dimensional chain structure. Moverover, via weak C9—H9C···O1 hydrogen bonds and π-π interactions involving two neighbouring chains [the nearest atom-to-atom distance between neighbouring imidazole rings is 3.405 (5) Å], a tubular polymer structure, propagating in [100], is formed.

### Experimental

The title compund was synthesized according to the reported procedure (Metobo *et al.*, 2006). Colourless plate-like crystals, suitable for X-ray diffraction, were obtained by slow evaporation of a solution of the title compound in Diethyl ether at room temperature.

### Refinement

H atoms were placed geometrically and refined as riding atoms: C—H = 0.96 Å (CH_3_) and 0.93 Å (CH), with U_iso_(H) = k × U_eq_(C), where k = 1.5 for (CH_3_), and = 1.2 for (CH) H-atoms.

### Crystal data

**Table:**

C_9_H_12_N_2_O_3_	*V* = 511.7 (8) Å^3^
*M**_r_* = 196.21	*Z* = 2
Triclinic, *P*1	*F*(000) = 208
Hall symbol: -P 1	*D*_x_ = 1.274 Mg m^−^^3^
*a* = 5.972 (3) Å	Mo *K*α radiation, λ = 0.71073 Å
*b* = 7.173 (7) Å	Cell parameters from 360 reflections
*c* = 12.164 (11) Å	µ = 0.10 mm^−^^1^
α = 79.630 (16)°	*T* = 293 K
β = 86.620 (15)°	Plate, colourless
γ = 89.326 (15)°	0.14 × 0.11 × 0.03 mm

### Data collection

**Table:**

Bruker APEX CCD area detector diffractometer	1769 independent reflections
Radiation source: fine-focus sealed tube	915 reflections with *I* > 2σ(*I*)
graphite	*R*_int_ = 0.022
φ and ω scans	θ_max_ = 25.0°, θ_min_ = 1.7°
Absorption correction: multi-scan (*SADABS*; Bruker, 2001)	*h* = −7→7
*T*_min_ = 0.987, *T*_max_ = 0.997	*k* = −8→8
2633 measured reflections	*l* = −14→8

### Refinement

**Table:**

Refinement on *F*^2^	Primary atom site location: structure-invariant direct methods
Least-squares matrix: full	Secondary atom site location: difference Fourier map
*R*[*F*^2^ > 2σ(*F*^2^)] = 0.062	Hydrogen site location: inferred from neighbouring sites
*wR*(*F*^2^) = 0.149	H-atom parameters constrained
*S* = 0.99	*w* = 1/[σ^2^(*F*_o_^2^) + (0.0655*P*)^2^] where *P* = (*F*_o_^2^ + 2*F*_c_^2^)/3
1769 reflections	(Δ/σ)_max_ < 0.001
130 parameters	Δρ_max_ = 0.17 e Å^−^^3^
0 restraints	Δρ_min_ = −0.16 e Å^−^^3^

### Special details

**Table:**

Geometry. All esds (except the esd in the dihedral angle between two l.s. planes) are estimated using the full covariance matrix. The cell esds are taken into account individually in the estimation of esds in distances, angles and torsion angles; correlations between esds in cell parameters are only used when they are defined by crystal symmetry. An approximate (isotropic) treatment of cell esds is used for estimating esds involving l.s. planes.
Refinement. Refinement of *F*^2^ against ALL reflections. The weighted *R*-factor wR and goodness of fit *S* are based on *F*^2^, conventional *R*-factors *R* are based on *F*, with *F* set to zero for negative *F*^2^. The threshold expression of *F*^2^ > σ(*F*^2^) is used only for calculating *R*-factors(gt) etc. and is not relevant to the choice of reflections for refinement. *R*-factors based on *F*^2^ are statistically about twice as large as those based on *F*, and *R*- factors based on ALL data will be even larger.

### Fractional atomic coordinates and isotropic or equivalent isotropic displacement parameters (Å^2^)

**Table:**

	*x*	*y*	*z*	*U*_iso_*/*U*_eq_	
O1	0.3982 (5)	0.3453 (4)	0.8094 (2)	0.0910 (9)	
O2	0.3540 (3)	0.2118 (3)	0.27957 (17)	0.0501 (6)	
O3	−0.0065 (4)	0.2329 (4)	0.34612 (19)	0.0794 (9)	
N1	0.5369 (4)	0.2729 (4)	0.5822 (2)	0.0562 (8)	
N2	0.2860 (4)	0.2475 (4)	0.4569 (2)	0.0480 (7)	
C1	0.3197 (5)	0.2938 (4)	0.6277 (3)	0.0505 (9)	
C2	0.5087 (5)	0.2466 (4)	0.4808 (3)	0.0503 (9)	
H2	0.6262	0.2289	0.4301	0.060*	
C3	0.1660 (5)	0.2779 (4)	0.5522 (3)	0.0529 (9)	
H3	0.0110	0.2859	0.5627	0.063*	
C4	0.2649 (7)	0.3323 (5)	0.7404 (3)	0.0668 (11)	
H4	0.1142	0.3480	0.7606	0.080*	
C5	0.1920 (6)	0.2290 (5)	0.3551 (3)	0.0519 (9)	
C6	0.3044 (5)	0.1971 (5)	0.1619 (3)	0.0470 (8)	
C7	0.5376 (5)	0.1878 (5)	0.1061 (3)	0.0634 (10)	
H7A	0.6202	0.2993	0.1119	0.095*	
H7B	0.5257	0.1806	0.0286	0.095*	
H7C	0.6144	0.0775	0.1426	0.095*	
C8	0.1737 (5)	0.0162 (5)	0.1638 (3)	0.0702 (11)	
H8A	0.2552	−0.0899	0.2017	0.105*	
H8B	0.1532	−0.0001	0.0884	0.105*	
H8C	0.0299	0.0243	0.2023	0.105*	
C9	0.1821 (5)	0.3734 (5)	0.1092 (3)	0.0662 (11)	
H9A	0.0359	0.3771	0.1460	0.099*	
H9B	0.1674	0.3714	0.0312	0.099*	
H9C	0.2654	0.4835	0.1168	0.099*	

### Atomic displacement parameters (Å^2^)

**Table:**

	*U*^11^	*U*^22^	*U*^33^	*U*^12^	*U*^13^	*U*^23^
O1	0.117 (2)	0.095 (2)	0.0680 (19)	−0.0003 (17)	−0.0229 (17)	−0.0287 (16)
O2	0.0374 (12)	0.0691 (16)	0.0448 (14)	−0.0015 (10)	0.0004 (10)	−0.0143 (12)
O3	0.0360 (14)	0.139 (3)	0.0686 (18)	0.0020 (13)	−0.0019 (12)	−0.0330 (17)
N1	0.0523 (18)	0.064 (2)	0.0522 (19)	−0.0018 (13)	−0.0066 (14)	−0.0074 (15)
N2	0.0376 (15)	0.0592 (19)	0.0468 (18)	−0.0004 (12)	−0.0004 (14)	−0.0089 (14)
C1	0.057 (2)	0.046 (2)	0.049 (2)	−0.0062 (16)	0.0026 (18)	−0.0079 (17)
C2	0.0387 (18)	0.055 (2)	0.055 (2)	−0.0014 (15)	−0.0029 (16)	−0.0051 (19)
C3	0.0437 (19)	0.060 (2)	0.054 (2)	−0.0038 (16)	0.0089 (18)	−0.0105 (19)
C4	0.085 (3)	0.054 (3)	0.062 (3)	−0.007 (2)	−0.005 (2)	−0.013 (2)
C5	0.040 (2)	0.064 (2)	0.051 (2)	−0.0007 (16)	0.0038 (18)	−0.0114 (18)
C6	0.0434 (18)	0.058 (2)	0.041 (2)	0.0034 (16)	−0.0065 (15)	−0.0121 (17)
C7	0.047 (2)	0.086 (3)	0.057 (2)	−0.0012 (18)	0.0030 (17)	−0.016 (2)
C8	0.055 (2)	0.074 (3)	0.086 (3)	−0.0117 (19)	−0.0017 (19)	−0.026 (2)
C9	0.067 (2)	0.068 (3)	0.063 (3)	0.0089 (19)	−0.0101 (19)	−0.007 (2)

### Geometric parameters (Å, °)

**Table:**

O1—C4	1.206 (4)	C4—H4	0.9300
O2—C5	1.315 (4)	C6—C9	1.512 (4)
O2—C6	1.501 (4)	C6—C8	1.518 (5)
O3—C5	1.196 (3)	C6—C7	1.519 (4)
N1—C2	1.301 (4)	C7—H7A	0.9600
N1—C1	1.398 (4)	C7—H7B	0.9600
N2—C3	1.376 (4)	C7—H7C	0.9600
N2—C2	1.378 (4)	C8—H8A	0.9600
N2—C5	1.417 (4)	C8—H8B	0.9600
C1—C3	1.356 (4)	C8—H8C	0.9600
C1—C4	1.464 (5)	C9—H9A	0.9600
C2—H2	0.9300	C9—H9B	0.9600
C3—H3	0.9300	C9—H9C	0.9600
			
C5—O2—C6	121.3 (2)	C9—C6—C8	113.1 (3)
C2—N1—C1	104.5 (3)	O2—C6—C7	102.4 (2)
C3—N2—C2	106.1 (3)	C9—C6—C7	110.8 (3)
C3—N2—C5	125.2 (3)	C8—C6—C7	111.5 (3)
C2—N2—C5	128.7 (3)	C6—C7—H7A	109.5
C3—C1—N1	110.6 (3)	C6—C7—H7B	109.5
C3—C1—C4	124.5 (3)	H7A—C7—H7B	109.5
N1—C1—C4	124.9 (3)	C6—C7—H7C	109.5
N1—C2—N2	112.7 (3)	H7A—C7—H7C	109.5
N1—C2—H2	123.6	H7B—C7—H7C	109.5
N2—C2—H2	123.6	C6—C8—H8A	109.5
C1—C3—N2	106.1 (3)	C6—C8—H8B	109.5
C1—C3—H3	126.9	H8A—C8—H8B	109.5
N2—C3—H3	126.9	C6—C8—H8C	109.5
O1—C4—C1	125.7 (4)	H8A—C8—H8C	109.5
O1—C4—H4	117.2	H8B—C8—H8C	109.5
C1—C4—H4	117.2	C6—C9—H9A	109.5
O3—C5—O2	129.1 (3)	C6—C9—H9B	109.5
O3—C5—N2	121.5 (3)	H9A—C9—H9B	109.5
O2—C5—N2	109.4 (3)	C6—C9—H9C	109.5
O2—C6—C9	109.5 (2)	H9A—C9—H9C	109.5
O2—C6—C8	109.0 (3)	H9B—C9—H9C	109.5

### Hydrogen-bond geometry (Å, °)

**Table:**

*D*—H···*A*	*D*—H	H···*A*	*D*···*A*	*D*—H···*A*
C2—H2···O3^i^	0.93	2.36	3.251 (5)	160
C9—H9C···O1^ii^	0.96	2.65	3.531 (5)	153

Symmetry codes: (i) *x*+1, *y*, *z*; (ii) −*x*+1, −*y*+1, −*z*+1.
